# Addition of Two Substantial Side-Branch Silencers to the Interference Silencer by Incorporating a Zero-Mass Metamaterial

**DOI:** 10.3390/ma15155140

**Published:** 2022-07-24

**Authors:** Shuichi Sakamoto, Juung Shin, Shota Abe, Kentaro Toda

**Affiliations:** 1 Department of Engineering, Niigata University, Ikarashi 2-no-cho 8050, Nishi-ku, Niigata 950-2181, Japan; 2 Graduate School of Science and Technology, Niigata University, Ikarashi 2-no-cho 8050, Nishi-ku, Niigata 950-2181, Japan; t16m034a@gmail.com (J.S.); t16m902a@gmail.com (S.A.); f21b105g@gmail.com (K.T.)

**Keywords:** zero-mass metamaterial, transmission loss, interference silencer, side-branch silencer

## Abstract

Zero-mass metamaterials comprise an orifice and a thin film. The resonance between the film and the air mass of the orifice hole is caused by sound waves, which significantly decreases the transmission loss at a specific frequency. The study novelly incorporates acoustic metamaterials in the delay tube of an interference silencer. In this case, it is determined that an interference silencer and a “side-branch silencer with two different branch pipe lengths” can be realized in a single silencer. At certain frequencies, the acoustic mass of the acoustic metamaterial approaches zero, which results in an interference silencer with the full length of the delay tube applied. At other frequencies, the acoustic metamaterial acts as a rigid wall with high transmission loss, thereby reflecting sound waves at the zero-mass metamaterial location. In this case, it is a side-branch silencer with two different tube lengths, corresponding to the tube lengths from the entrance and exit of the delay tube to the zero-mass metamaterial, respectively. The incorporation of zero-mass metamaterial into an interference-type silencer can introduce the silencing effect of a side-branch silencer with two different branch tube lengths without increasing the volume of the interference-type silencer. Theoretical values were obtained using the transfer matrix. Consequently, the theoretical and experimental values were close, enabling us to predict the transmission loss of the proposed silencer.

## 1. Introduction

In an interference silencer, the peak frequency at which the sound reduction effect can be obtained is uniquely determined by the delay tube length. Thus, the use of multiple delay tubes, depending on the required sound reduction frequency, increases the silencer volume. Therefore, we focused on zero-mass metamaterials [1], in which the acoustic mass approaches zero at a specific frequency. 

Acoustic metamaterials are new artificial materials that have been developed in the last few decades. In addition, theoretical analysis development [2] has shown the potential for further new performance and various applications in practical engineering fields [3,4]. For example, it can provide a noise-free environment in certain bandwidths [5] or allow near-zero density operation by connecting tubular waveguides with ultranarrow tubes [6]. Zero-mass metamaterials comprise an orifice and a thin film. The resonance between the film and the air mass of the orifice hole is caused by sound waves, which significantly decreases the transmission loss at a specific frequency [7]. Recently, more complex designs of acoustic metamaterials with membranes have been reported to improve their acoustic performance. For example, holes can be perforated in the membrane [8] to introduce the Helmholtz resonator effect, or constrained structures [9] can be used to suppress certain eigenmodes of the membrane. Moreover, active control can be introduced to tune the acoustic performance [10].

The study novelly incorporates acoustic metamaterials in the delay tube of an interference silencer. In this case, it is determined that an interference silencer and a “side-branch silencer with two different branch pipe lengths” can be realized in a single silencer.

Herein, an overview of the interference silencer with built-in acoustic metamaterials is proposed. At certain frequencies, the acoustic mass of the acoustic metamaterial approaches zero, which results in an interference silencer with the full length of the delay tube applied. At other frequencies, the acoustic metamaterial acts as a rigid wall with high transmission loss, thereby reflecting sound waves at the zero-mass metamaterial location. In this case, it is a side-branch silencer with two different tube lengths, corresponding to the tube lengths from the entrance and exit of the delay tube to the zero-mass metamaterial, respectively.

In the theoretical analysis, the transfer matrix method [11,12] was employed for the interference-type silencer ducts. For the zero-mass metamaterial, the acoustic impedance was calculated using the Equation of motion for a spring-mass (damper) system in which the thin film and air in the orifice hole have the same mass and behave similarly. The acoustic impedance was integrated into the transfer matrix of the pipeline system of the silencer. The transmission loss was calculated for the transfer matrix of the silencer and compared with the experimental results to verify the sound reduction characteristics.

## 2. Measuring Equipment and Samples

### 2.1. Transmission Loss Measurement

**Figure 1:**
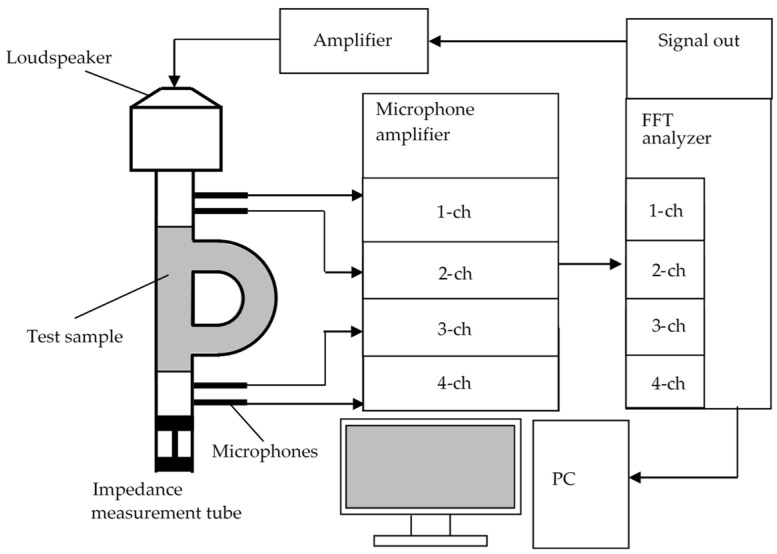
Schematic of the impedance measurement tube and measurement equipment.

The transmission loss was measured using a Brüel & Kjær Type 4206T 4-microphone impedance-measuring tube (Nærum, Denmark) and an Ono Sokki 4-channel-fast Fourier transform (FFT) analyzer DS-3000 (Yokohama, Japan). A schematic of the measurement apparatus is presented in Figure 1. The silencer is placed in a predetermined position, and two microphones are mounted each in front of and behind the sample. A signal generator and amplifier generate sound waves with a reference signal (linear multisine waves). Furthermore, the microphones detect the sound pressure before and after transmission through the sample. In addition, a 4-channel FFT analyzer measures the cross-spectrum via a signal amplifier for the microphones. The transmission loss was calculated in accordance with the American Society for Testing and Materials (ASTM) E2611-09.

### 2.2. Zero-Mass Metamaterial and Delay Tube of Interferometric Silencer 

**Figure 2:**
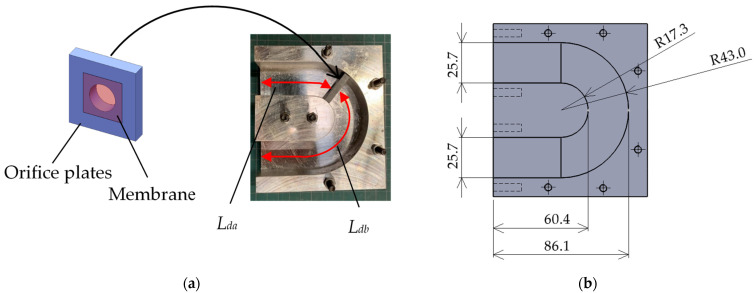
(**a**) Delay tube section of the interference silencer with incorporated zero-mass metamaterial; (**b**) dimensions of the delay tube.

Figure 2a presents the delay tube section of the interference silencer with zero-mass metamaterials used herein. Figure 2b presents the dimensions of the delay tube. Table 1 and Table 2 present the specifications of the thin films and orifice plates used in the experiments, respectively.

Herein, a 25.7 mm^2^ orifice plate fabricated by a photo fabrication type 3D printer, Formlabs Form2 (Somerville, MA, USA), was used, and photocuring resins were used. The membrane material was attached and fixed to the orifice plate coated with Vaseline without tension.

The delay tube of the interference silencer used herein was fabricated from an A5052 aluminum alloy. Table 3 shows the chemical composition of the delay tube of the interference silencer used in the experiments. The inner cross-section of the delay tube was a square of 25.7 mm side, and the length of the centerline of the delay tube was 180.9 mm. The delay tube length (*L_d_*) was divided into the upstream (*L_da_*) and downstream (*L_db_*) lengths, respectively, at the position where the zero-mass metamaterial was installed. The main tube length (*L_m_*) was 60.4 mm.

**Figure 3:**
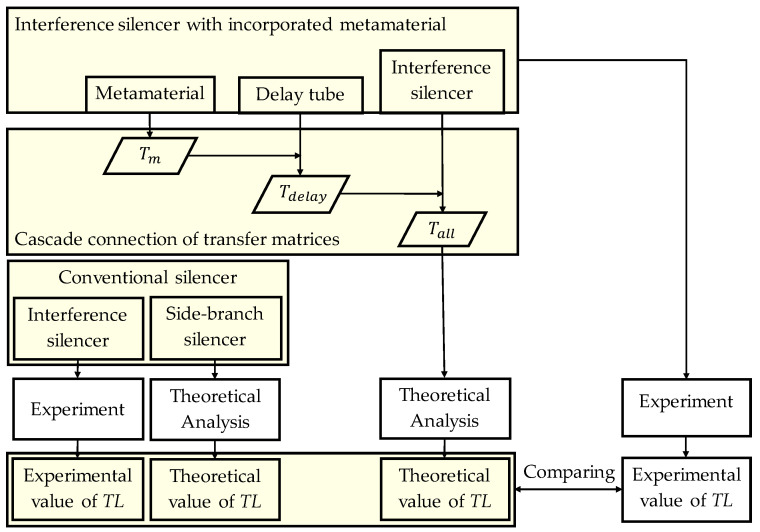
Flowchart of experimental and theoretical analysis process.

The flow of the subsequent experimental and theoretical analysis is shown in the flowchart in Figure 3. For comparison, the sound reduction characteristics of a conventional interference silencer are described in Section 3.

## 3. Theoretical Analysis

### 3.1. Analysis of Zero-Mass Metamaterial 

**Figure 4:**
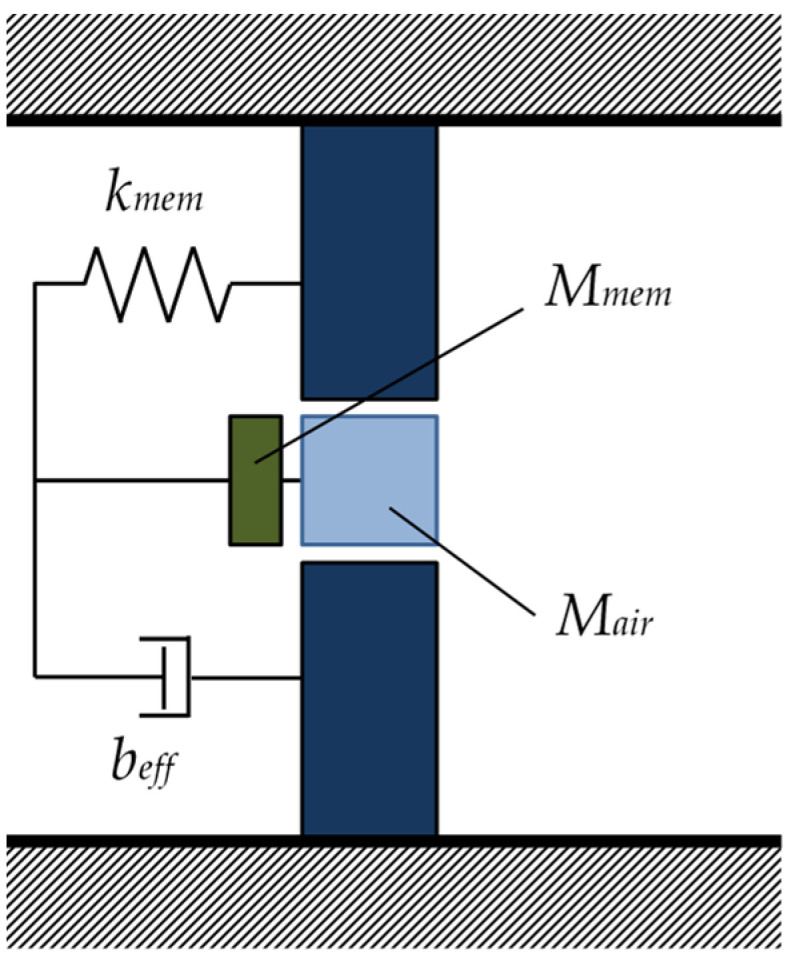
Analytical model for zero-mass metamaterials.

The zero-mass metamaterial comprises a thin film attached to an orifice plate. Figure 4 presents an analytical model of the zero-mass metamaterial. Herein, the calculation is performed as a one-degree-of-freedom vibration system in which the film and the air in the orifice hole vibrate identically. *M_mem_* denotes the mass of the membrane attached to the orifice hole; *M_air_*, the mass of the air in the orifice hole; *k_mem_*, the spring constant of the membrane; and *b_eff_*, the damping coefficient of the zero-mass metamaterial. Here, the spring constant of the thin film *k_mem_* was measured using the method of Lee et al. [13].

In addition, *t* is defined as the orifice plate thickness; *r*, the orifice hole radius; and *λ*, the wavelength of the incident sound wave. Since *r* << *λ*, the zero-mass metamaterial is assumed to be a concentrated element model.

**Figure 5:**
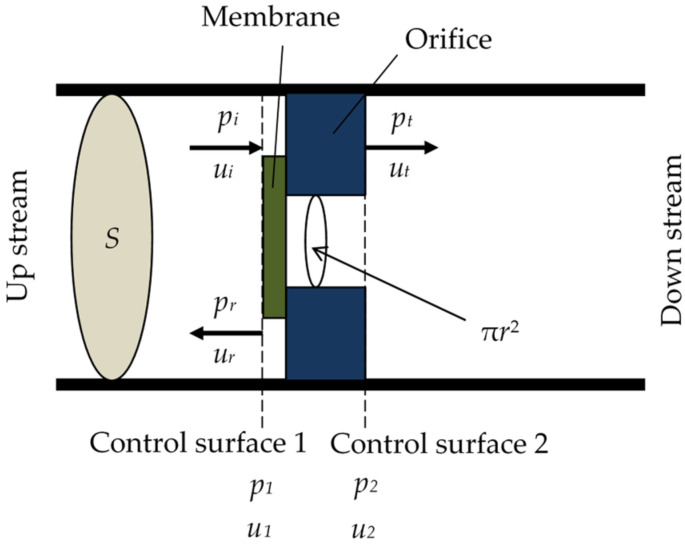
Variable name near zero-mass metamaterial.

The names of the variables near the zero-mass metamaterial are presented in Figure 5. Notably, *p_i_* is the incident sound pressure; *p_r_*, the reflected sound pressure; *p_t_*, the transmitted sound pressure; *u_i_*, the incident particle velocity; *u_r_*, the reflected particle velocity; and *u_t_*, the transmitted particle velocity. Furthermore, *S* is the cross-sectional area of the acoustic tube. Note that *p_1_* and *u_2_* are given by Equations (1)–(4) expressing *p_2_* and *u_2_*, respectively.

(1) $$p_{1}=p_{i}+p_{r}$$

(2) $$u_{2}=u_{i}+u_{r}$$

(3) $$p_{2}=p_{t}$$

(4) $$u_{2}=u_{t}$$

If *t* and *r* are sufficiently smaller than *λ*, the membrane and air at the orifice hole are considered to oscillate in unison as described above [1,13], and the particle velocities before and after the zero-mass metamaterial are equal. This scenario is expressed in Equation (5).

(5) $$u_{1}=u_{2}$$

Here, using the characteristic impedance (*Z_0_*) of air, the sound pressure can be expressed as Equations (6)–(8), respectively.

(6) $$p_{i}=Z_{0}u_{i}$$

(7) $$p_{r}=-Z_{0}u_{r}$$

(8) $$p_{t}=Z_{0}u_{t}$$

The relationship between the sound pressure, particle velocity, and transfer matrix is expressed by Equation (9).

(9) $$\left[\begin{array}{c} p_{1}\\ Su_{1} \end{array}\right]=\left[\begin{array}{cc} T_{11} & T_{12}\\ T_{21} & T_{22} \end{array}\right]\left[\begin{array}{c} p_{2}\\ Su_{2} \end{array}\right]$$

From a previous study on zero-mass metamaterials [1], the Equation of motion for this structure is given by Equation (10), where *ξ* is the displacement of the membrane and air at the orifice hole, and $\xi \left(t\right)=\xi_{0}\exp\left(-i\omega t\right)$.

(10) $$\left(p_{1}-p_{2}\right)\pi r^{2}=M_{eff}\ddot{\xi }+b_{eff}\dot{\xi }$$

Here, *M_eff_* denotes the effective mass defined by Equation (11), and *b_eff_* is the effective damping coefficient of the zero-mass metamaterial. To simplify the calculations, *b_eff_* is set to zero because its effect on the theoretical value of transmission loss is negligible.

(11) $$M_{eff}=M_{air}+M_{mem}-\frac{k_{mem}}{\omega^{2}}$$

Reorganizing Equation (10) using Equations (2) and (4), Equation (12) is obtained [1].

(12) $$p_{i}+p_{r}-p_{t}=\left(\frac{1}{\pi r^{2}}\right)\left(-i\omega M_{eff}+b_{eff}\right)\ddot{\xi }$$

Reorganizing the above Equations using Equations (6) and (7), Equation (13) is obtained.

(13) $$2p_{i}-2p_{t}=p_{t}\left(\frac{S}{Z_{0}\pi^{2}r^{4}}\right)\left(b_{eff}-i\omega M_{eff}\right)$$

Substituting Equation (13) into Equation (9) using Equations (1) and (4), Equation (14) is obtained.

(14) $$\left[\begin{array}{c} p_{1}\\ Su_{1} \end{array}\right]=\left[\begin{array}{cc} 1 & \frac{S}{\pi^{2}r^{4}}\left(b_{eff}-i\omega M_{eff}\right)\\ 0 & 1 \end{array}\right]\left[\begin{array}{c} p_{2}\\ Su_{2} \end{array}\right]$$

Thus, the transfer matrix *T_m_* of the zero-mass metamaterial is given by Equation (15).

(15) $$T_{m}=\left[\begin{array}{cc} 1 & \frac{S}{\pi^{2}r^{4}}\left(b_{eff}-i\omega M_{eff}\right)\\ 0 & 1 \end{array}\right]$$

### 3.2. Theoretical Analysis of Delay Tube with Built-In Zero-Mass Metamaterials 

**Figure 6:**
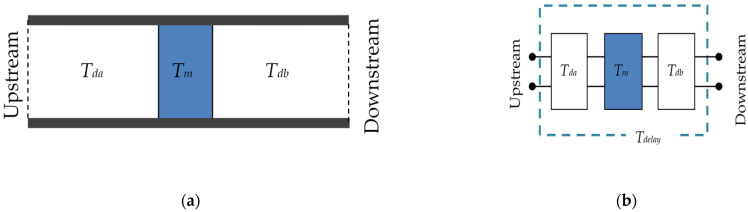
Acoustic elements of the delay tube: (**a**) acoustic elements of the delay tube and (**b**) equivalent circuit of the delay tube.

Figure 6 presents the acoustic elements of the delay tube section and equivalent circuit corresponding to the acoustic system. In Figure 6, the tube elements are upstream and downstream of the zero-mass metamaterial and are connected by a cascade connection. The cascade-connected transfer matrix *T* can be expressed as in Equation (16) using *T_m_* in Equation (15).

(16) $$T_{delay}=T_{f}T_{m}T_{b}=\left[\begin{array}{cc} A & B\\ C & D \end{array}\right]$$

Here, the transfer matrix for the upstream tube element of the metamaterial is presented in Equation (17), using air density *ρ_0_* and speed of sound *c_0_* in the air.

(17) $$T_{f}=\left[\begin{array}{cc} \cos kl_{f} & i\frac{\rho_{0}c_{0}}{S}\sin kl_{f}\\ i\frac{S}{\rho_{0}c_{0}}\sin kl_{f} & \cos kl_{f} \end{array}\right]$$

Similarly, the transfer matrix for the downstream tube element is presented in Equation (18).

(18) $$T_{b}=\left[\begin{array}{cc} \cos kl_{b} & i\frac{\rho_{0}c_{0}}{S}\sin kl_{b}\\ i\frac{S}{\rho_{0}c_{0}}\sin kl_{b} & \cos kl_{b} \end{array}\right]$$

### 3.3. Transfer Matrix for Entire Interference Silencer

Here, *k* denotes the wavenumber; *l_f_*, the length of the upstream delay tube; *l_b_*, the length of the downstream delay tube; and *S*, the cross-sectional area of the main and delay tubes. If the transfer matrices *T_delay_* and *T_2_* are expressed by Equation (19), *T* after a parallel connection is expressed by Equation (20).

(19) $$T_{delay}=\left[\begin{array}{cc} A_{1} & B_{1}\\ C_{1} & D_{1} \end{array}\right],\;T_{2}=\left[\begin{array}{cc} A_{2} & B_{2}\\ C_{2} & D_{2} \end{array}\right]\;$$

(20) $$T=\left[\begin{array}{cc} \frac{A_{1}B_{2}+A_{2}B_{1}}{B_{1}+B_{2}} & \frac{B_{1}B_{2}}{B_{1}+B_{2}}\\ C_{1}+C_{2}+\frac{\left(A_{2}-A_{1}\right)\left(D_{1}-D_{2}\right)}{B_{1}+B_{2}} & \frac{D_{1}B_{2}+D_{2}B_{1}}{B_{1}+B_{2}} \end{array}\right]\;$$

**Figure 7:**
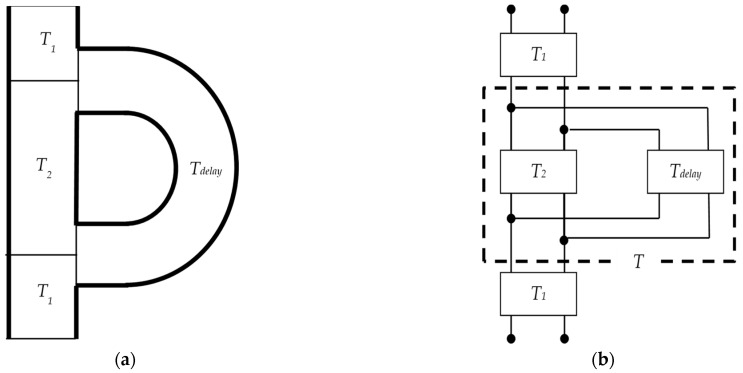
Acoustic elements and equivalent circuit of an interference silencer: (**a**) division into acoustic elements and (**b**) equivalent circuit.

The cross-sectional views of the entire interference silencer and the equivalent circuit corresponding to its acoustic system are presented in Figure 7. In addition, *T_1_*-, *T*-, and *T_1_*-connected cascades are *T_all_*, and are expressed by Equation (21).

(21) $$T_{all}=T_{1}TT_{1}=\left[\begin{array}{cc} A_{all} & B_{all}\\ C_{all} & D_{all} \end{array}\right]\;$$

Using the four-terminal constants *A_all_*–*D_all_* in Equation (21), the transmission loss (*TL*) of the interference silencer can be expressed as in Equation (22).

(22) $$TL=10\log_{10}\frac{{\left|A_{all}+\frac{S}{\rho_{0}c_{0}}B_{all}+\frac{\rho_{0}c_{0}}{S}C_{all}+D_{all}\right|}^{2}}{4}\;$$

The delay tube is connected perpendicular to the side of the main tube. An opening end correction must be added to the tube end of the delay tube with respect to the geometric tube length. Many studies have been conducted on aperture end correction [14,15]. Because these tubes are equal in the cross-sectional area, a value of 0.8 was used for the aperture end correction value [15]. Therefore, the aperture end correction length was 0.8 times the equivalent circular radius of the 25.7 mm^2^ aperture.

### 3.4. Peak Frequency of Sound Attenuation for Ordinary Interference-Type Silencers and Side-Branch Silencers 

For comparison with the silencer proposed herein, the sound reduction characteristics of the conventional interference and side-branch silencers are described.

The phase change amount in the main and delay tubes of the interference-type silencer can be expressed as Equations (23) and (24), respectively.

(23) $$\theta_{m}=\frac{2\pi fL_{m}}{c_{0}}\;$$

(24) $$\theta_{d}=\frac{2\pi fL_{d}}{c_{0}}\;$$

When the difference between these two-phase changes satisfies Equation (25), the sound waves propagating in the main and delay tubes merge in the opposite phase at the confluence and are canceled.

(25) $$\left|\theta_{m}-\theta_{d}\right|=\left(2n-1\right)\pi \;\left(n=1,\;2,\;3\ldots \right)$$

The above Equation can be rearranged so that the peak frequency of sound attenuation of an interference-type silencer can be expressed by Equation (26).

(26) $$f_{peak}=\left|\frac{\left(2n-1\right)c_{0}}{2\left(L_{m}-L_{d}\right)}\right|\;\left(n=1,\;2,\;3\ldots \right)\;$$

The peak frequency of the side-branch silencer can be expressed as in Equation (27), where *L_side_* denotes the length of the branch tube of the side-branch silencer.

(27) $$f_{side}=\left|\frac{\left(2n-1\right)c_{0}}{4L_{side}}\right|\;\left(n=1,\;2,\;3\ldots \right)\;$$

In an interference-type silencer, the peak attenuation culminates at the frequency *f_peak_* satisfying Equations (28)–(31) and its odd-numbered multiples [16]. Therefore, it is sufficient to determine the delay tube length of the interference-type silencer so that the “dip frequency of the *TL* of the zero-mass metamaterial in the interference-type silencer” and the “peak frequency of the sound reduction of the interference-type silencer” coincide.

The dip frequency of the *TL* of the zero-mass metamaterial used herein is 1425 Hz; thus, the acoustic mass of the acoustic metamaterial approaches zero at ~1425 Hz in the proposed silencer. Therefore, it is necessary to select the total delay tube length such that the peak frequency of sound reduction is ~1425 Hz.

(28) $$L_{d}-L_{m}=\frac{\lambda }{2}\;$$

(29) $$L_{d}+L_{m}=\lambda \;$$

(30) $$L_{d}=\frac{3c}{4f}\;$$

(31) $$L_{m}=\frac{c}{4f}\;$$

The peak sound attenuation frequencies of the side-branch silencers corresponding to the two branch tube lengths divided by the zero-mass metamaterial are presented in Table 4.

## 4. Experimental and Theoretical Values for Transmission Loss 

### 4.1. Transmission Loss of Single Zero-Mass Metamaterial

The zero-mass metamaterial comprises an orifice and a thin film. The orifice plate has a hole diameter *d* of 10 mm and a thickness *t* of 5 mm, and the film is made of low-density polyethylene with a thickness *t_m_* of 11 μm.

**Figure 8:**
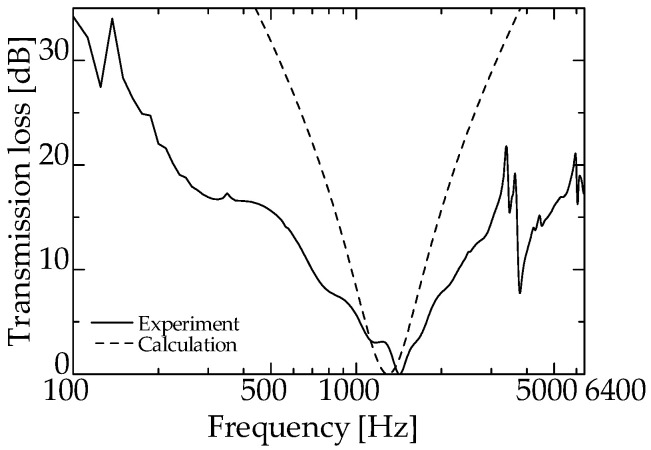
Transmission loss of single zero-mass metamaterial (experimental and theoretical).

Figure 8 presents the experimental and theoretical values for the zero-mass metamaterial. Evidently, sound waves penetrate the zero-mass metamaterial at a dip frequency of 1425 Hz.

In addition, sound waves are considered to be reflected in the frequency band where the *TL* is significant.

### 4.2. Comparison of Experimental and Theoretical Values

A comparison of the theoretical and experimental values based on the theoretical analysis described in the previous section is shown.

**Figure 9:**
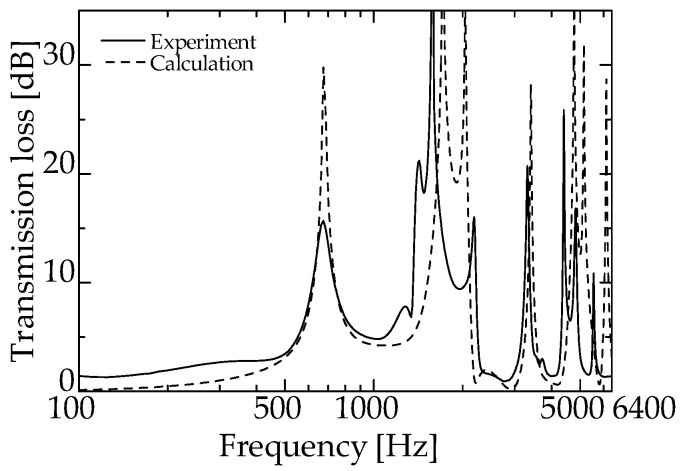
Experimental and theoretical values of the interference silencer with incorporated zero-mass metamaterial (zero-mass metamaterials installed at *L_da_* = 50 mm).

**Figure 10:**
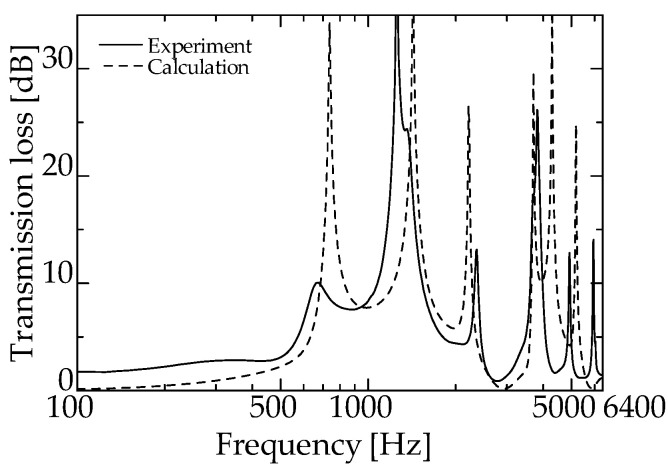
Experimental and theoretical values of the interference silencer with incorporated zero-mass metamaterial (zero-mass metamaterials installed at *L_da_* = 60 mm).

Figure 9 and Figure 10 present the experimental and theoretical values for zero-mass metamaterials installed at *L_da_* = 50 and 60 mm, respectively.

As can be seen from Figure 8, the dip frequency of the *TL* of the integrated zero-mass metamaterial is 1425 Hz, indicating that for the proposed silencer, the acoustic mass of the acoustic metamaterial approaches zero at a specific frequency, i.e., around 1425 Hz. Therefore, the total length of the delay tube of the interference silencer was set so that the peak frequency of sound reduction is ~1425 Hz.

Figure 9 and Figure 10 present the experimental and theoretical sound attenuation peaks that are roughly consistent with the designed attenuation frequency of 1425 Hz for an interference silencer with *L_d_* = 180.9 mm (Figure 9 and Figure 10). Therefore, it can be assumed that the acoustic mass of the zero-mass metamaterial approaches zero as expected.

The difference in the peak sound attenuation magnitude and frequency between the experimental and theoretical values is probably because the sound waves incident on the zero-mass metamaterial is incompletely transmitted at the dip frequency of the *TL* and are incompletely reflected at the frequency of the high *TL*.

**Figure 11:**
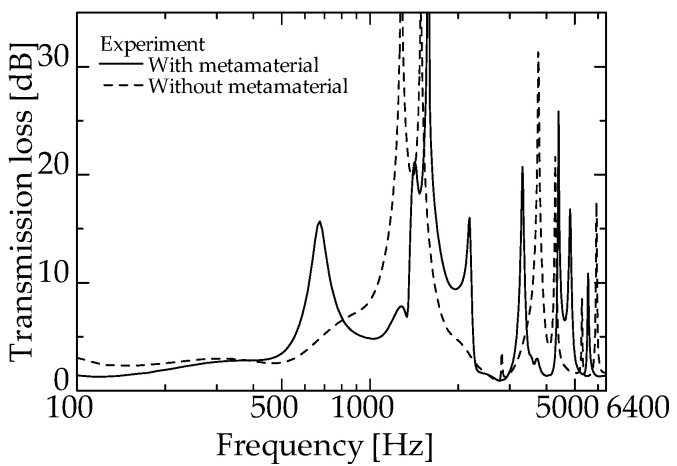
Comparison of the experimental values with and without zero-mass metamaterial (zero-mass metamaterials at *L_da_* = 50 mm).

**Figure 12:**
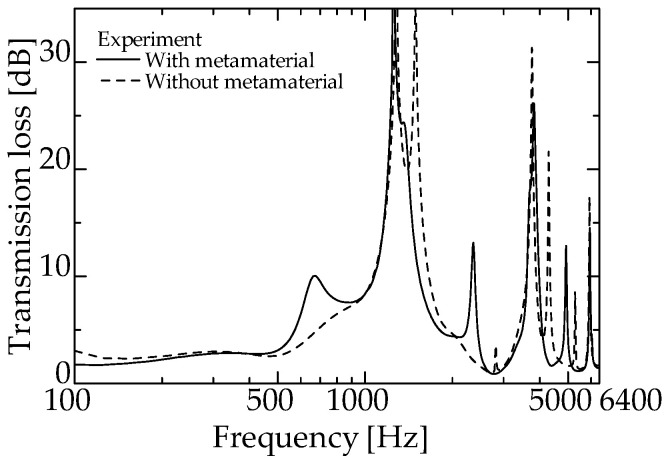
Comparison of the experimental values with and without zero-mass metamaterial (zero-mass metamaterials at *L_da_* = 60 mm).

Figure 11 and Figure 12 present the experimental values of an ordinary interference silencer and the experimental values of a zero-mass metamaterial at *L_da_* = 50 and 60 mm, respectively (redrawn from Figure 9 and Figure 10).

For both experimental values in Figure 11, a sound reduction peak is observed near 1425 Hz. This frequency band is the dip frequency band of the zero-mass metamaterial (Figure 7). The same is true for Figure 12.

These facts suggest that the proposed silencer worked similarly to an ordinary interference silencer. Thus, the delay tube with zero-mass metamaterials acted like the delay tube of an ordinary interference-type silencer. The above results confirm that the acoustic mass of the zero-mass metamaterial is near zero around the dip frequency presented in Figure 8.

**Figure 13:**
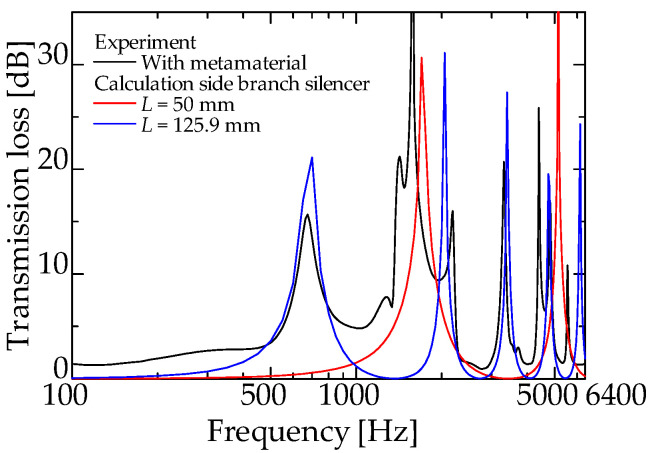
Comparison with simple side-branch silencer (experimental: interference silencer with zero-mass metamaterial; theoretical: side-branch silencer with 50 mm and 125.9 mm branch tube length).

Figure 13 shows the theoretical values for two different conventional side-branch silencers with *L_d_* = 50 and 125.9 mm, respectively, and the experimental values for a zero-mass metamaterial installed at *L_da_* = 50 mm (redrawn from Figure 13).

In Figure 13, the experimental values of the proposed silencer exhibit sound attenuation peaks at 675, 1700, and 2000 Hz, in addition to the attenuation peak of the interference silencer at 1425 Hz. The sound reduction peaks at 675 and 2000 Hz correspond to the first-order, third-order, and subsequent sound reduction peak frequencies (blue line in Figure 13) of the side-branch silencer with a 125.9 mm branch tube length. The experimental peak of the proposed silencer at 1700 Hz corresponds to the 1700 Hz peak of the side-branch silencer with a 50 mm branch tube length (red line in Figure 13).

**Figure 14:**
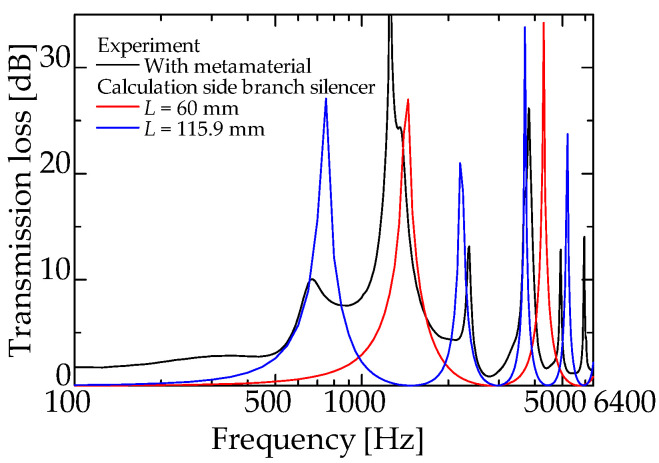
Comparison with simple side-branch silencer (experimental: interference silencer with zero-mass metamaterial; theoretical: side-branch silencer with 60 mm and 115.9 mm branch tube length).

Figure 14 presents the theoretical values for two types of conventional side-branch silencers, with *L_d_* = 60 and 115.9 mm, respectively, and the experimental values for a zero-mass metamaterial installed at *L_da_* = 60 mm (redrawn from Figure 14). Exactly as in Figure 13, the experimental values of the proposed silencer exhibit sound reduction peaks at 735, 1420, and 2200 Hz, in addition to the sound reduction peak near 1425 Hz of the interference silencer. Similarly, the 735 and 2200 Hz sound reduction peaks correspond to the respective peak frequencies of the side-branch silencer with a 115.9 mm branch tube length (blue line in Figure 14). The 1420 Hz peak of the proposed silencer corresponds to the 1420 Hz peak of the side-branch silencer with a 60 mm branch tube length (red line in Figure 14).

The above results indicate that the zero-mass metamaterial installed in the delay tube acts as a rigid wall with high *TL* at frequencies other than the specified frequency. Furthermore, the delay tube is divided into two closed-end tubes with two different tube lengths, acting as two side-branch silencers. These results confirm that the zero-mass metamaterial has an acoustically significant mass in the bands other than the dip frequency of the zero-mass metamaterial.

## 5. Conclusions

Herein, the performance of a silencer with zero-mass metamaterial built into the delay tube of an interference-type silencer was evaluated via theoretical analysis of TL and experiments, and the following conclusions were obtained:

1. The incorporation of zero-mass metamaterial into an interference-type silencer can introduce the silencing effect of a side-branch silencer with two different branch tube lengths without increasing the volume of the interference-type silencer. Consequently, the total volume of the interference and two side-branch silencers could be reduced by half.
2. The incorporation of zero-mass metamaterials in the interference silencer increased the sound reduction peaks.
3. Theoretical analysis of the interference-type silencer with built-in zero-mass metamaterial was conducted using the Equations of motion, and theoretical values were obtained using the transfer matrix. Consequently, the theoretical and experimental values were close, enabling us to predict the *TL* of the proposed silencer.

## Figures and Tables

**Table 1:** Specifications of thin film material.

Material of Membrane	Nominal Density (kg/m^3^)	Measured Membrane Thickness (μm)
Low-density polyethylene	920	11

**Table 2:** Specifications of orifice plate.

Material of Orifice	Thickness of Plate (mm)	Diameter of Hole (mm)
Photocurable resin	5	10

**Table 3:** Chemical composition of A5052 aluminum alloy.

Element	Ratio (%)	Element	Ratio (%)
Aluminum	Balance	Copper	0.10 max
Magnesium	2.2–2.8	Manganese	0.10 max
Chromium	0.15–0.35	Zinc	0.10 max
Silicon	0.25 max	Others, each	0.05 max
Iron	0.40 max	Others, total	0.15 max

**Table 4:** Peak frequency of the side-branch silencer sound attenuation at each branch tube length.

		**Length of Side-Branch Tube** ***L_side_*** **(mm)**			
		50.0	60.0	125.9	115.9
Peak Frequency *f_side_* (Hz)	*n* = 1	1701.5	1418.0	675.7	734.0
	*n* = 2	5104.5	4253.8	2027.2	2202.1

## Nomenclature

ASTM	American Society for Testing and Materials
*A-D, A_all_–D_all_*	Four-terminal constants
*b_eff_*	Damping coefficient
*c_0_*	Speed of sound in the air (m/s)
*d*	Orifice plate diameter (mm)
*f, f_peak_, f_side_*	Peak frequency (Hz)
FFT	Fast Fourier transform
*I*	Imaginary unit
*k*	Wavenumber
*k_mem_*	Spring constant of the thin film (N/m)
*L_d_, L_da_, L_db_, L_m_, L_side_*	Length of the tube (mm)
*M_air_*	Mass of the air in the orifice hole
*M_eff_*	Effective mass
*M_mem_*	Mass of the membrane attached to the orifice hole
*p*_1_, *p*_2_, *p_i_*, *p_r_*, *p_t_*	Sound pressure (Pa)
*r*	Orifice hole radius (mm)
*S*	Cross-sectional area of the acoustic tube (mm^2^)
*t*	Orifice plate thickness (mm)
*t_m_*	Film thickness (mm)
*T, T_1_*, *T_2_*, *T_11_*, *T_12_*, *T_21_*, *T_22_*, *T_all_*, *T_b_*, *T_delay_*, *T_f_*, *T_m_*	Transfer matrix
*TL*	Transmission loss (dB)
*u*_1_, *u*_2_, *u_i_*, *u_r_*, *u_t_*	Particle velocity (m/s)
